# Components of the antepartum, intrapartum, and postpartum exposome impact on distinct short-term adverse neonatal outcomes of premature infants: A prospective cohort study

**DOI:** 10.1371/journal.pone.0207298

**Published:** 2018-12-05

**Authors:** Unzila Ali Nayeri, Catalin S. Buhimschi, Guomao Zhao, Irina A. Buhimschi, Vineet Bhandari

**Affiliations:** 1 Department of Obstetrics, Gynecology and Reproductive Sciences, Yale University, School of Medicine, New Haven, Connecticut, United states of America; 2 Department of Pediatrics, Yale University, School of Medicine, New Haven, Connecticut, United states of America; University of Oklahoma, UNITED STATES

## Abstract

We aimed to test the hypothesis that determinants of the perinatal clinical exposome related to the underlying etiology of premature birth (PTB) impact differently on select neonatal outcomes. We conducted a prospective longitudinal study of 377 singleton preterm neonates [gestational age (GA) at birth: 23–34 weeks] separated into three distinct contemporaneous newborn cohorts: i) spontaneous PTB in the setting of intra-amniotic infection/inflammation (yes-IAI, n = 116); ii) spontaneous PTB in the absence of IAI (no-IAI, n = 130), and iii) iatrogenic PTB for preeclampsia (iPTB-PE, n = 131). Newborns (n = 372) were followed until death or discharge. Amniotic fluid defensins 1&2 and calgranulins A&C were used as biomarkers of IAI. An algorithm considering cord blood interleukin-6 (IL-6) and haptoglobin (Hp switch-on) was used to assess fetal exposure to IAI. Intraventricular hemorrhage (IVH), periventricular leukomalacia (PVL), necrotizing enterocolitis (NEC), bronchopulmonary dysplasia (BPD), retinopathy of prematurity (ROP), early-onset neonatal (EONS) and late-onset (LOS) sepsis, death. Independent risk factors for adverse outcomes were: i) IVH (n = 53): histologic chorioamnionitis, GA, fetal growth restriction, male sex, Hp switch-on; ii) PVL (n = 11): cord blood IL-6; iii) NEC (n = 25), GA; iv) BPD (n = 53): ventilator support, need for surfactant, GA; v) ROP (n = 79): ventilator support, Hp switch-on, GA; vi) fetal and neonatal death (n = 31): GA, amniotic fluid IL-6; vii) suspect EONS (n = 92): GA, Hp switch-on; viii) LOS (n = 81): GA. Our findings are applicable to pregnancies delivered between 23 and 34 weeks’ gestation in the setting of IAI and PE, and suggest that GA and inflammatory intrauterine environment play key roles in occurrence of IVH, PVL, ROP, death, EONS and LOS. Postnatal determinants seem to play major role in NEC and BPD.

## Introduction

Premature birth (PTB), defined as birth prior to 37 weeks gestation, remains a significant public health problem [1]. In the U.S., the current PTB rate is 9.9%, representing an increase over the last few years [2]. The rate of very-low birthweight newborns (<1,500 grams) remained unchanged at 1.4% [2]. Very premature newborns are at the highest risk of early postnatal death or short-term neonatal complications including intraventricular hemorrhage (IVH), necrotizing enterocolitis (NEC), bronchopulmonary dysplasia (BPD), retinopathy of prematurity (ROP), and periventricular leukomalacia (PVL) [3]. Presence and severity of any of these short-term complications in preterm survivors are significant risk factors for neurodevelopmental delay and long-term disability [4–5].

Out of all PTBs, 70% occur consequent to spontaneous onset of uterine contractions or preterm prelabor rupture of fetal membranes (PPROM) [6]. Provider-initiated PTBs (30–35%) are most often due to concern for preeclampsia (PE), fetal growth restriction (FGR), or both [7–8].Gestational age (GA) and birth weight are correlated with short-term neonatal outcomes [9]. However, contribution of the exposome, a concept collectively describing the role of environment on impacting the disease outcome based on its interaction with the subject's genetic make-up, remains largely unknown [10–11]. The preterm newborn exposome differs from that of a term baby in terms of antenatal, intrapartum, and postnatal exposures. As a result, the exposome may influence the structural and functional integrity of the fetus and the clinical progress of the premature newborn. Inflammatory processes associated with spontaneous PTB in the setting of intra-amniotic infection are known to adversely affect the fetus [12–15]. There is compelling evidence that complications of fetal organ systems (respiratory, gastrointestinal, immune, central nervous system) that are characteristic of early-onset neonatal sepsis (EONS) are at least partially triggered by inflammatory processes *in utero* [12,16]. It is not well established how the intensity of the fetal inflammatory response in conjunction with GA and other antenatal (e.g. steroids, magnesium sulfate, acid-base status, antibiotics, FGR) and postnatal (e.g. antibiotics, surfactant, ventilator support) factors affect the occurrence of various neonatal complications. Physicians’ ability to detect, at birth presence of biomarkers indicative of neonatal susceptibility and of biological response to an environmental challenge, will represent a significant step forward. Haptoglobin (Hp) is likely to add to our understanding why some but not all premature newborns delivered in the context of infection-induced PTB have poor outcomes [17].

The determinism of prematurity resulting from iatrogenic PTB is different from that of spontaneous PTL or PPROM [18–19]. From this perspective, for the same GA at birth, a fetus exposed to intra-amniotic infection/inflammation (IAI, Triple-I) may have a higher risk of IVH, but not NEC, which could be more frequently encountered by neonates of PE mothers [20]. Our hypothesis was that antenatal, intra-partum and post-partum clinical and environmental factors are linked to neonatal outcomes in a differential fashion. Our objective was to identify determinants of the perinatal clinical exposome related to the underlying etiology of PTB that impact differently on short-term neonatal outcomes.

## Materials and methods

### Population and study design

We studied 378 consecutive preterm singleton newborns born to mothers who delivered preterm between 23–34 weeks of gestation and consented previously to enrollment in our study. Women were recruited following admission to Labor and Birth or to the High Risk antepartum units at Yale New Haven Hospital (YNHH) and followed prospectively until delivery. Data collection for this study lasted 60 months. Inclusion criteria were symptoms of spontaneous preterm labor or PPROM requiring a clinically indicated amniocentesis to rule out IAI, or severe PE necessitating iatrogenic PTB. Maternal signs and symptoms for which an amniocentesis was indicated to rule-out IAI included fever (>100.4°F), persistent abdominal contractions unrelieved by hydration, abdominal tenderness, and advanced cervical dilatation (>3 cm). All newborns were admitted to YNHH Newborn Intensive Care Unit (NICU) and were followed prospectively until death or discharge. Indications for delivery, maternal and neonatal medical care or admission were based on the judgment of the clinicians independent of our research protocol. To verify the accuracy of our data we conducted periodic audits through random sampling of cases by two investigators and one research nurse. The audits showed excellent agreement of case reports. None of the enrolled patients withdrew from the study, and follow-up data were available for all neonates admitted to the NICU. The Yale University Human Investigation Committee approved the study protocol. Written informed consent was obtained from all study participants.

**Fig 1:**
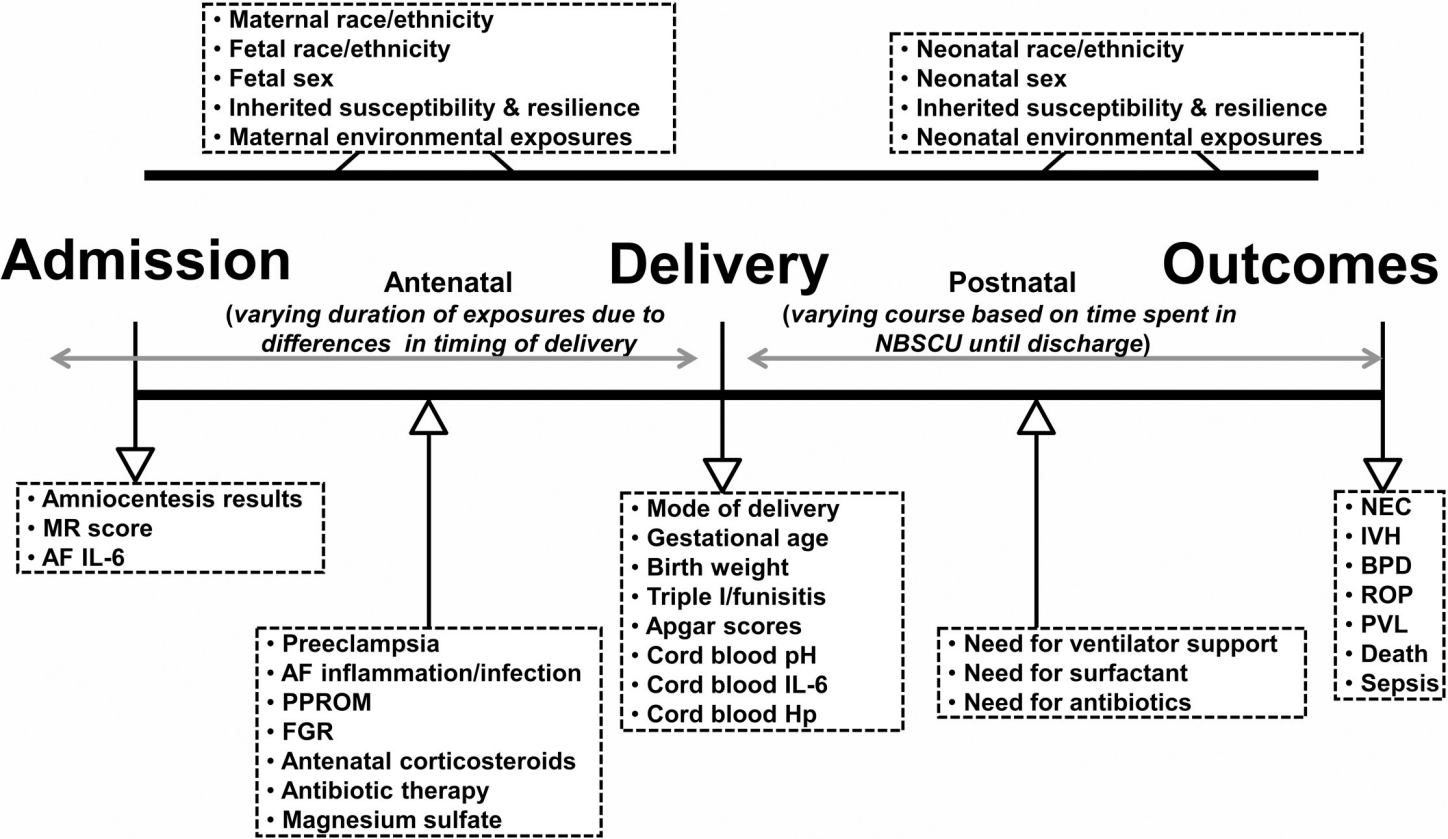
Schematic representation of fetal and neonatal exposome based on exposures and events that may occur in the antepartum, intrapartum, and postpartum periods. Clinical and laboratory variables informative of the exposome are listed inside the boxes. *Abbreviations*: NBSCU, newborn special care unit; IVH, intraventricular hemorrhage; NEC, necrotizing enterocolitis; BPD, bronchopulmonary dysplasia; ROP, retinopathy of prematurity; PVL, periventricular leukomalacia; MR, mass restricted; AF, amniotic fluid; PPROM, preterm premature rupture of membranes; FGR, fetal growth restriction; Triple I, intra-amniotic infection/inflammation; Hp, haptoglobin.

Three distinct contemporaneous cohorts of premature newborns emerged: **i)** preterm neonates born in the setting of spontaneous PTL or PPROM and evidence of intra-amniotic infection/inflammation (Yes-IAI, n = 116); **ii)** preterm neonates born in the setting of spontaneous PTL or PPROM absent IAI at the time of the amniocentesis (No-IAI, n = 131); **iii)** premature newborns delivered iatrogenic for maternal and/or fetal indications in the context of PE (n = 131). The PE group was the best option we had to compare the results of neonates delivered in the context of spontaneous PTB vs. iatrogenic induced PTB where the potential of antenatal exposure of the baby to IAI was minimal. Performance of the amniocentesis procedure as well as the recommendation for delivery occurred independent of our study protocol. Primary study outcomes were incidence of IVH, NEC, BPD, ROP, PVL, and/or postnatal death. Baseline demographic (sex, race, GA), antenatal (magnesium sulfate, steroids, antibiotics, membrane status, amniotic fluid infection, histologic chorioamnionitis [HCA], FGR, and postnatal (ventilator support, surfactant, sepsis) variables were collected at or near the time of delivery and used to identify the strongest model predictive of each adverse neonatal outcome. An important feature of our study was availability of the NICU derived data collected in the YNHH Newborn Special Care unit on a real-time basis. Fig 1 depicts a schematic representation of the study design and variables used to characterize the antenatal, intrapartum, and postnatal exposome, respectively.

### Clinical definitions

GA was established based on last menstrual period confirmed by an ultrasonographic examination prior to 20 weeks [21]. PTB was defined as delivery of the neonate <37 weeks GA [22]. At our institution, an amniocentesis was not clinically indicated to diagnose IAI in women presenting with symptoms of preterm labor or PPROM after >34 weeks. Thus, the upper range of GA was 34 weeks for all study groups. PE was defined according to established clinical and laboratory criteria [23]. FGR was defined as ultrasonographic estimated fetal growth less than the 10^th^ percentile [24].

EONS was defined as confirmed or suspected sepsis ≤72 h after birth [25]. Neonatal hematological indices and sepsis categorization were assessed from neonatal blood specimens and microbial cultures obtained within 2 h from birth [26]. Late-onset sepsis (LOS) was defined by positive blood cultures >72 h after birth. Details are provided in the S1 Appendix. IVH was defined according to the Papile’s classification of cranial ultrasound (routinely done at least twice in the first 10 days of life, and later as clinically indicated) by findings of blood in the germinal matrix or ventricular system, with or without ventricular dilation and parenchymal extension [27]. NEC was defined according to Bell’s classification as stage II and above [28]. BPD was defined as the need for supplemental oxygen at 36 weeks postnatal age along with characteristic radiographic changes [29]. ROP was defined as per the international classification [30]. Presence of PVL was considered based on cerebral ultrasound findings of increased echogenicity and cystic lesions in the periventricular white matter [31].

### Biological samples

For the No-IAI and Yes-IAI groups of newborns, amniotic fluid was available for analysis in all cases per study design. In a subset of PE cases (41/131), for research purpose only, amniotic fluid was retrieved in sterile fashion trans-myometrial at the time of Cesarean delivery prior to delivery of the baby. Umbilical cord gas analysis was performed from venous and arterial cord blood samples. Excess umbilical vein blood was collected for research purpose and was available in 84% (97/116), 72% (93/130) and 68% (89/131) of Yes-IAI, No-IAI and PE groups cases, respectively. Technical details about preparation and storage of the biological materials are provided in the S1 Appendix.

### Clinical diagnosis and research evaluation of IAI and HCA

Clinical laboratory tests performed to diagnose IAI included glucose, lactate dehydrogenase (LDH), Gram stain, and white blood cell (WBC) count. Microbiological analysis of amniotic fluid included Gram stain and microbial cultures. In all 378 cases, placenta was evaluated and HCA was graded as previously described [32–33]. Details are provided in the S1 Appendix.

For research purposes, presence of IAI was assessed by proteomic profiling of amniotic fluid as previously described [34–35]. As the 4 biomarkers of the MR score (defensin-1, defensin-2, calgranulin-A, calgranulin-C) are part of the innate immune response of the amniotic cavity, classification of IAI using this method reflects the host response to environmental exposure (presence of microorganisms or other noxious stimuli capable able of eliciting a host response) [10,17]. Presence in the amniotic fluid of 3–4 of the above mentioned biomarkers was indicative of IAI.

### Interleukin-6 (IL-6) immunoassays

IL-6 levels were measured in amniotic fluid and cord serum by ELISA (Pierce-Endogen, Rockford, IL) by investigators unaware of sample origin (see S1 Appendix).

### Assessment of fetal exposure to IAI by Hp switch-on status

In prior publications, we demonstrated that the ability of the fetus to precociously switch-on Hp as an adaptive host response can serve as marker of antenatal exposure to IAI [10,17]. Absence of cord blood Hp at the protein level denotes Hp0-0 phenotype (anhaptoglobinemia). A detectable Hp β-chain (42 kDa) on Western blot was indicative of a “switch-on” (see S1 Appendix).

### Statistical analysis

Statistical analyses were performed using SigmaPlot, (v12 Systat, Chicago, IL) and MedCalc (Broekstraat, Belgium) statistical software programs. Normality testing was performed using the Shapiro-Wilk test. Data was presented as median and interquartile ranges as appropriate. Mann-Whitney or Kruskal-Wallis ANOVA tests followed by multiple comparison procedures (Dunn’s method) were performed as appropriate. Comparisons between proportions were done with Chi square or Fisher exact tests. A stepwise logistic regression analysis was used to determine concurrent relationships between variables and to identify the strongest model predictive of each adverse neonatal outcome. The model included as independent variables maternal and neonatal admission variables, GA at birth, route of delivery, sex, steroids, exposure to magnesium for neuroprotection, and need for surfactant. Model quality was assessed using the Hosmer-Lemeshow goodness-of-fit test [36]. A backwards approach was taken to select those variables that associated most strongly with neonatal morbidity. We calculated the sample size based on a 10% likelihood difference between rates of neonatal adverse outcomes if the neonates are exposed to IAI. Assuming 80% power and a 2-sided α = 0.05, at least 110 patients were needed in each group. A *P* value of <0.05 was considered significant throughout the analysis.

## Results

### Characteristics of women

Table 1 displays the demographic and clinical characteristics of women and newborns enrolled in this study. Women in the No-IAI group were more likely to be white, compared to Yes-IAI and PE groups, where the proportion of African American women was higher (*P*<0.001). Women with PTB in the setting of Yes-IAI were enrolled and delivered at earlier GAs compared with both other groups (*P*<0.001). Newborns of both Yes-IAI and No-IAI groups were more likely to be exposed to ruptured membranes and prenatal antibiotic therapy (*P*<0.001). As expected, pregnancies delivered in the setting of PE had a higher frequency of antenatal magnesium sulfate exposure and FGR (*P*<0.001). All 6 stillbirths in our study occurred in the PE group (*P* = 0.003). The prevalence of provider initiated deliveries and lower birthweights was higher in women in Yes-IAI and PE groups (P<0.001). The length of stay in the NICU was significantly higher for neonates delivered by mothers with Yes-IAI or PE compared to those of mothers with No-IAI. Among live born newborns, those in the Yes-IAI group had a higher proportion of Apgar scores <7 at 5 min compared with the other two groups (*P* = 0.009). Babies born in the context of PE were more often delivered by Cesarean for indications such as breech presentation or induction failure (*P*<0.001).

**Table 1:** Clinical characteristics of women and newborns.

Variable	MATERNAL PTB GROUPS			*P* value
	No-IAI	Yes-IAI	PE	
	n = 131	n = 116	n = 131	
***Maternal and antepartum characteristics***				
Age, *years* *	28 [24–33]	28 [22–34]	30 [24–35]	0.322
Gravidity *	2 [1–4]	3 [2–4]	2 [1–4]	0.404
Parity *	1 [0–1]	0 [0–2]	1 [0–2]	0.408
Maternal race/ethnicity †				
Non-Hispanic white	69 (53)	29 (25)	42 (33)	<0.001
African American	38 (29)	54 (47)	58 (44)	
Hispanic	17 (13)	26 (22)	24 (18)	
Other	7 (5)	7 (6)	7 (5)	
Gestational age, *weeks* *	29.6 [26.3–31.6]	27.2 [24.7–29.6]	29.2 [27–31.3]	<0.001
Ruptured membranes †	74 (56)	60 (52)	0 (0)	<0.001
Steroid exposure during pregnancy †	122 (93)	110 (95)	121 (92)	0.742
Prenatal antibiotic treatment †	104 (79)	97 (84)	7 (5)	<0.001
Antenatal magnesium sulfate exposure †	47 (36)	46 (40)	122 (93)	<0.001
FGR †	2 (2)	0 (0)	37 (28)	<0.001
***Delivery and newborn characteristics***				
Provider-initiated delivery † ‡	45 (34)	82 (71)	131 (100)	<0.001
Gestational age at delivery, *weeks* *	30.6 [28.1–32.5]	27.4 [25.1–30.1]	29.5 [27.1–31.6]	<0.001
Cesarean delivery †	61 (47)	47 (41)	115 (88)	<0.001
Stillbirth †	0 (0)	0 (0)	6 (5)	0.003
Newborn sex †§				
Female	72 (55)	49 (42)	57 (46)	0.112
Male	59 (45)	67 (58)	68 (54)	
Birthweight, *grams* *§	1,600 [1,120–1,980]	1,000 [773–1,453]	1,098 [810–1,436]	<0.001
NICU, days *	28.0 [14–60]	49.5 [23–97]	40.0 [21–71]	<0.001
Apgar score at 1 min †§	7 [5–8]	7 [4–8]	7 [4–8]	0.500
Apgar score at 5 min †§	9 [8–9]	8 [7–9]	8 [7–9]	0.059
Apgar score at 5 min <7 ‡§	13 (10)	28 (25)	18 (14)	0.009

* Data presented as median [interquartile range] and analyzed by Kruskal-Wallis ANOVA

† Data presented as n (%) and analyzed by Chi square tests

‡ Refers to an approach where the pregnancy was no longer managed expectantly

§ Variable reported only for live born newborns.

Abbreviations: FGR, fetal growth restriction; NICU, Neonatal Intensive Care Unit; IAI, intra-amniotic infection/inflammation; PE, preeclampsia

### Amniotic fluid, placenta, and cord blood analytes

In Table 2 we present the results of the laboratory evaluations of amniotic fluid, placenta, and umbilical cord blood.

**Table 2:** Laboratory and Histological analyses.

Variable	MATERNAL PTB GROUPS			*P* value
	No-IAI	Yes-IAI	PE	
***Amniotic fluid***	***n = 131***	***n = 116***	***n = 41*** ^***#***^	
Glucose, *mg/dL* *	27 [19–37]	4 [2–13]	NA	<0.001
LDH, *U/L* *	176 [122–254]	744 [442–1,790]	NA	<0.001
WBC, *cells/mm*^*3*^ *	7 [2–16]	947 [222–2,63]	NA	<0.001
Positive Gram stain †	12 (9)	62 (55)	NA	<0.001
Positive cultures †	17 (13)	79 (70)	NA	<0.001
Positive Gram stain or culture	21 (16)	85 (75)	NA	<0.001
Mass Restricted (MR) score *	0 [0–2]	4 [3–4]	0 [0–1]	<0.001
Interleukin-6 (pg/mL) ‡, §	798 [246–2,796]	29,214 [9,69–77,244]	102 [43–230]	<0.001
***Placental pathology***	***n = 130***	***n = 115***	***n = 126*** ^***#***^	
Chorionic plate inflammation, *stage* ‡	0 [0–2]	3 [2–3]	0 [0–0]	<0.001
Amnionitis, grade ‡	0 [0–1]	3 [2–3]	0 [0–0]	<0.001
Funisitis, grade ‡	0 [0–0]	2 [0–4]	0 [0–0]	<0.001
Decidual hemorrhage/abruption †	18 (14)	18 (16)	32 (26)	0.035
Maternal HCA ║	0 [0–1]	2 [1–2]	0 [0–0]	<0.001
Fetal HCA ¶	0 [0–2]	2 [2–2]	0 [0–0]	<0.001
***Cord blood analysis***	***n = 94***	***n = 97***	***n = 90*** ^***#***^	
Interleukin 6 (pg/mL) ‡ §	7 [6–68]	31 [9–214]	3 [3–5]	<0.001
Haptoglobin (ng/mL) ‡ §	429 [9–6,157]	8,002 [974–11,899]	1,005 [517–2,776]	<0.001
Haptoglobin switch-on pattern †§	29 (31)	67 (69)	6 (7)	<0.001
Arterial pH ‡	7.31 [7.27–7.33]	7.32 [7.29–7.35]	7.25 [7.17–7.28]	<0.001
Arterial base deficit ‡	4.3 [2.3–6.3]	4.8 [3.6–7.1]	5.9 [3.5–10]	0.010
Venous pH ‡	7.36 [7.33–7.39]	7.37 [7.34–7.40]	7.28 [7.23–7.31]	<0.001
Venous base excess ‡	3.4 [1.6–5.2]	3.9 [2.0–5.3]	4.9 [2.6–7.8]	0.031

* Data presented as median [interquartile range] and analyzed by Mann-Whitney tests. *MR scoring*: MR 0: “no” inflammation; MR 1–2: “minimal” inflammation; MR 3–4: “severe” inflammation).

† Data presented as n (%) and analyzed by Chi square tests.

‡ Data presented as median [interquartile range] and analyzed by Kruskal-Wallis ANOVA.

§ Variable used exclusively for research purposes.

║ Maternal HCA (histologic chorioamnionitis) is defined as: amnionitis grade 0 = 0 (absent), amnionitis grades 1–2 = 1 (mild), amnionitis grades 3–4 = 2 (severe); *Ghidini et al*. *Obstet & Gynecol*. *2000;96(2)*:*201–206*.

¶ Fetal HCA (histologic chorioamnionitis) is defined as: chorionic place 0 and funisitis 0 = 0 (absent); chorionic plate I-II and funisitis grades 1–2 = 1 (mild); chorionic plate III and funisitis grades 3–4 = 2 (severe); *Ghidini et al*. *Obstet & Gynecol*. *2000;96(2)*:*201–206*.

Abbreviations: WBC, white blood cell count; LDH, lactate dehydrogenase

# All newborns with reported values were live born.

Women in the Yes-IAI group had lower amniotic fluid glucose levels, higher LDH, elevated WBCs, and positive Gram stain and/or amniotic fluid cultures (*P*<0.001 for all). In the Yes-IAI group, all women had MR scores 3–4 as grouped by study design, and this associated with markedly elevated concentration of amniotic fluid IL-6 (*P*<0.001). Interestingly, several samples from the No-IAI group grew bacteria, but the MR score was 0 (zero), implying contamination or sampling prior to the onset of IAI. Women who underwent intra-operative amniocentesis during Cesarean delivery in the context of PE had extremely low levels of amniotic fluid IL-6, significantly lower than women in No-IAI and Yes-IAI groups (*P*<0.001). In patients with Yes-IAI, placental histopathology confirmed higher severity of inflammation in all placental compartments (*P*<0.001 for all). Cord blood IL-6 and Hp levels were significantly higher in fetuses of women in the Yes-IAI group. Similarly, the frequency of Hp switch-on status was significantly higher in the Yes-IAI group, implying fetal host response to antenatal exposure to inflammation. In the cohort of pregnancies complicated by PE, both cord blood arterial and venous pH values were significantly lower compared with the other two groups (*P*<0.001).

### Predictors of short-term neonatal complications

**Fig 2:**
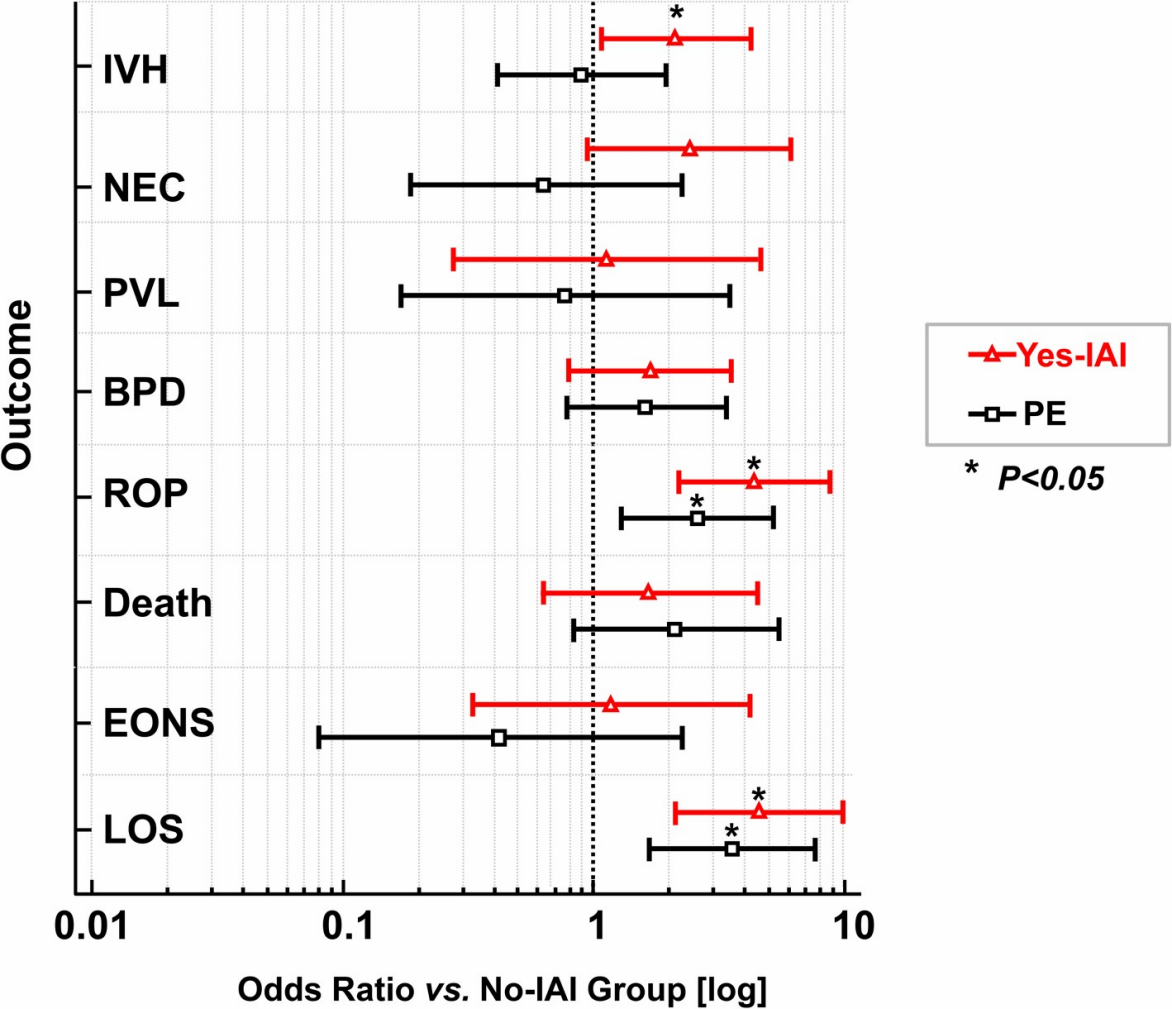
Forest plot of odds ratios of postnatal outcomes in newborns exposed to intra-amniotic inflammation (Yes-IAI) or preeclampsia (PE). Log odds ratios (open symbols) and 95% confidence intervals (horizontal lines) for outcomes relative to reference group (**No-IAI**). Vertical interrupted line marks the point of no difference from reference group. * Outcomes were significantly different (*P*<0.05). Abbreviations: IVH, intraventricular hemorrhage; NEC, necrotizing enterocolitis; BPD, bronchopulmonary dysplasia; ROP, retinopathy of prematurity; PVL, periventricular leukomalacia; EONS, early-onset neonatal sepsis; LOS, late-onset sepsis.

The frequency of adverse short-term neonatal outcome for each study group is displayed in Table 3. IVH, ROP, and late-onset sepsis occurred more often in newborns born in the setting of IAI, compared with No-IAI newborns. Newborns of preeclamptic mothers had significantly higher frequency of ROP and late-onset sepsis than No-IAI newborns. They also had lower frequency of NEC when compared to Yes-IAI newborns. Fig 2 illustrates the log odds ratios of individual short-term adverse neonatal outcomes for newborns born in the context of IAI or PE compared with the No-IAI group as referent.

**Table 3:** Frequency of short-term newborn outcomes in each study group.

Adverse outcome	Number of newborns completing evaluation	MATERNAL PTB GROUPS			*P* value
		No-IAI	Yes-IAI	PE	
IVH (grades 1–4) †	372				
Present		15 (11)	25 (22)	13 (10)	0.024
Absent		116 (89)	91 (78)	112 (90)	
NEC (grades 2–4) †	372				
Present		7 (5)	14 (12)	4 (3)	0.017
Absent		124 (95)	102 (88)	121 (97)	
PVL †	371				
Present		4 (3)	4 (3)	3 (2)	0.888
Absent		126 (97)	112 (97)	122 (98)	
BPD †	349				
Present		14 (13)	19 (18)	20 (19)	0.320
Absent		110 (87)	89 (82)	97 (81)	
ROP (grades 1–4) †	371				
Present		13 (10)	38 (33)	28 (22)	<0.001
Absent		117 (90)	78 (67)	97 (78)	
Fetal and neonatal death †	378				
Present		7 (5)	10 (9)	14 (11)	0.283
Absent		124 (95)	106 (91)	117 (89)	
EONS †	371				
Present		19 (15)	51 (44)	22 (18)	<0.001
Absent		112 (85)	65 (56)	102 (82)	
Confirmed EONS †	372				
Present		5 (4)	5 (4)	2 (2)	0.440
Absent		126 (96)	111 (96)	123 (98)	
Confirmed LOS †	361				
Present		10 (8)	30 (28)	29 (23)	<0.001
Absent		118 (92)	78 (72)	96 (77)	

† Data presented as n (%) and analyzed by Chi square tests.

Abbreviations: IVH, intra-ventricular hemorrhage; NEC, necrotizing enterocolitis; PVL, periventricular leukomalacia; BPD, bronchopulmonary dysplasia; ROP, retinopathy of prematurity; PTB, preterm birth; IAI, intra-amniotic inflammation/infection; PE, preeclampsia, EONS, early-onset neonatal sepsis; LOS, late-onset sepsis

Next, by using multivariable logistic regression analysis, we found that IVH was associated with HCA, GA at birth, FGR, male sex, and *in utero* exposure to inflammation as determined by cord blood Hp switch-on status (Table 4). The only predictor for NEC was GA at birth. Higher levels of cord blood IL-6 were an increased risk of PVL, implying an important role of fetal inflammation on this complication. Predictive factors for BPD included need for ventilator support, need for surfactant, and GA at delivery. Need for ventilator support, *in utero* exposure to IAI as determined by cord blood Hp switch-on status, and GA at birth were independent predictive factors of ROP. We found that decreasing GA at birth and IL-6 were significant predictors or stillbirth or neonatal death. GA and Hp switch-on status were significant determinant for EONS while LOS was significantly related to GA alone.

**Table 4:** Logistic regression analysis for risk of short-term newborn outcomes.

Adverse outcome, n	H-L	H-L	Significant independent predictors	Coefficient †	Standard error	Odds ratio	Odds ratio
	Goodness	*P* Value				[95%CI]	*P* Value
	of fit *						
IVH, n = 53	5.36	0.719	HCA	0.521	0.147	1.7 [1.3–2.2]	<0.001
			GA at birth	-0.265	0.061	0.8 [0.7–0.8]	<0.001
			FGR	1.597	0.557	4.9 [1.6–14.7]	0.004
			Male sex	0.849	0.341	2.4 [1.2–4.5]	0.013
			Hp switch-on status	1.674	0.341	5.3 [1.7–16.9]	0.004
NEC, n = 25	8.21	0.315	GA at birth	-0.196	0.083	0.8 [0.7–1.0]	0.019
PVL, n = 11	5.78	0.672	Cord blood IL-6 (log)	0.792	0.223	2.3 [1.4–3.5]	<0.001
BPD, n = 53	4.23	0.836	Ventilator support	1.730	0.437	5.6 [2.4–13.3]	<0.001
			Need for surfactant	1.693	0.685	5.4 [1.4–20.8]	0.013
			GA at birth	-0.250	0.091	0.8 [0.6–0.9]	0.006
ROP, n = 79	6.35	0.607	Ventilator support	0.746	0.376	5.8 [1.9–17.0]	<0.001
			Hp switch-on status	1.129	0.551	3.1 [1.1–9.1]	0.040
			GA at birth	-0.570	0.081	0.6 [0.5–0.7]	0.001
Death, n = 31	2.43	0.965	GA at birth	-0.974	0.188	0.4 [0.3–0.5]	<0.001
			Amniotic fluid IL-6 (log)	-0.659	0.242	0.5 [0.3–0.8]	0.006
EONS, n = 92	4.89	0.769	Hp switch-on status	1.354	0.409	3.9 [1.7–8.7]	<0.001
			GA at birth	-0.221	0.062	0.8 [0.7–0.9]	<0.001
Confirmed EONS, n = 12	3.38	0.908	GA at birth	-0.296	0.109	0.7 [0.6–0.9]	0.007
Confirmed LOS, n = 81	3.86	0.869	GA at birth	-0.384	0.005	0.7 [0.6–0.8]	<0.001

*The Hosmer-Lemeshow (H-L) goodness-of-fit test is a statistic that measures the quality of the model. A small value resulting in *P* value approaching 1 signifies a good model with non-significant difference between the observed and estimated outcomes.

† Individual regression coefficients expressed in logits. A negative coefficient signifies an inverse relationship between predictor and respective outcome.

Abbreviations: IVH, intra-ventricular hemorrhage; NEC, necrotizing enterocolitis; PVL, periventricular leukomalacia; BPD, bronchopulmonary dysplasia; ROP, retinopathy of prematurity; EONS, early-onset neonatal sepsis; LOS, late-onset sepsis; HCA, histologic chorioamnionitis; GA, gestational age; FGR, fetal growth restriction; Hp, haptoglobin.

## Discussion

To determine the antenatal, intrapartum and postnatal factors that contribute to short-term morbidity of preterm newborns, we evaluated to the best of our ability clinical and laboratory variables informative of fetal and neonatal exposomes. We focused our attention on neonates delivered <34 weeks, because the early PTB rate (<34 weeks) remained unchanged during the last decade at 2.76% [2]. This group of neonates is important because the rates of short and long-term adverse outcomes are much higher for babies delivered early versus late preterm [37]. To understand the main risks associated with premature delivery of a baby exposed to an inflammatory insult we focused our attention on pregnancies complicated by IAI. Different from previous epidemiologic retrospective studies, we included markers of amniotic fluid and cord blood inflammation, indications for delivery, placental pathology, and postnatal events in addition to multiple clinical variables including GA at birth, birth weight, and antenatal exposure to pharmacologic interventions (e.g. steroids, antibiotics, and magnesium sulfate).

We found that neonatal mortality and morbidity increased with delivery at earlier GA [38]. This was not surprising as previous data has shown that each additional week of gestation confers survival benefit in neonates delivered from 26 to 32 weeks GA [22]. Furthermore, premature infants are more likely to require additional support for special needs during their lifetime compared to those infants delivered at 37–42 weeks GA [39,40]. This is relevant as developmental delays of preterm babies has previously been linked to NEC, IVH, PVL, and ROP, complications that occur more often with birth at earlier GA [41–44]. These epidemiologic findings bring into discussion a need for better understanding of the mechanisms through which neonatal biologic immaturity predisposes to these conditions. The answers to these questions are complex and largely unknown. For example, for newborns born in the context of PE, disturbances in the maternal and placental angiogenic growth factor system may impact development and maturation of the pulmonary and retinal micro-vascular apparatus. It is worth considering that GA at birth is an important variable of the robustness to which fetal and neonatal innate immunity can respond to microbial challenge, which in turn can impact susceptibility to sepsis [45]. In addition, inflammation, exposure to general anesthesia, postnatal mechanical ventilation, placement of central lines, and exposure to various medications could play synergistic roles [41,42,44]. Based on our data, we can conclude that in pregnancies complicated by IAI or PE, GA was an independent risk factor for all short-term neonatal complications. However, in the current study and contrary to what has been previously proposed, GA was not the only factor determining the outcome [11].

A hostile antenatal intra-uterine inflammatory environment represented by amniotic fluid infection, elevated amniotic fluid and cord blood IL-6 levels along with the fetal response to this environment, as reflected by Hp switch-on status, were significantly associated with IVH, PVL, ROP, death, and EONS. Because not all premature neonates have a heightened state of inflammation at birth, our observations have pathophysiologic and clinical implications. It seems reasonable to assume that in pregnancies managed expectantly, the fetus is initially isolated and protected against amniotic fluid infection and inflammation provided infection did not spread hematogenously [46]. The compartmentalization process, however, aimed at protecting fetus and mother in the initial phase of the process, can be overcome especially in the setting of amniotic fluid infection and HCA [47]. The observed distribution pattern of cord blood Hp expression supports the above assertion [17]. Yet, in addition to its value as a biomarker of antenatal exposure to inflammation, Hp may have direct beneficial roles. Hp inhibits lipid peroxidation and binds free hemoglobin, thereby potentially limiting damage [48,49]. This may explain why Hp could be of benefit in conditions with high oxidative stress and angiogenic factor imbalance, such as IVH and ROP [50]. From a clinical perspective, our results are highly relevant. Free hemoglobin released during brain hemorrhage is cytotoxic and causes neuronal damage. In animal models of intracerebral hemorrhage, genetically-induced ahaptoglobinemia or hypohaptoglobinemia result in inability to neutralize free hemoglobin leaked inside the brain, leading to severe brain injury and neuronal deficits [51,52]. Interestingly, we found that low cord blood IL-6 level was associated with stillbirth or neonatal death. Because most stillbirths were encountered in the PE group, we believe this phenomenon could be linked with the fetus’ inability to mount an inflammatory response. Our assertion is supported by previous studies demonstrating that babies of mothers with PE display disrupted hematopoiesis manifested as neonatal thrombocytopenia and neutropenia [53]. Because transcription of the human Hp gene is induced by IL-6, a fetal hypo-inflammatory response would be also associated with low Hp levels and death, as previously shown [50,54].

While attention of clinician scientists has traditionally focused on the postnatal relationship between oxygen needs and ROP, Lee and Dammann proposed that risk of ROP could be increased following *in utero* exposure to inflammation, in a “double-hit” theory [55]. Klinger et al. reported in a large cohort that EONS is a risk factor for ROP [56]. Interestingly, a previous report linked PE with ROP [57]. The underlying mechanism of this association remains unknown. It is possible that similar to the placenta, fetal retinal vasculature reacts to hypoxia with heightened stabilization of hypoxia inducible factor-1α (HIF-1α) [58]. Given the regulatory role of oxygen and involvement of HIF-1α in ROP, newborns born in the context of PE may also have higher tissue sensitivity to the toxic effects of oxygen with higher output of vascular endothelial growth factor (VEGF) [59]. Our analysis revealed that the risk of ROP in PE is twice that of No-IAI newborns, but lower than the risk in Yes-IAI neonates. As HIF-1α and VEGF upregulation could also result from exposure to an inflammatory process, this could also explain the heightened ROP risk in Yes-IAI newborns [56].

We found that in addition to antenatal exposure to inflammation, neonates that developed ROP had an increased need for ventilator support [60,61]. In premature babies, hyperoxia achieved during ventilation represents a well-recognized risk of ROP. Consequently, for many years practitioners focused their attention on the relationship between retinal vascular proliferation, VEGF, and high vs low oxygen saturation [62,63]. The search for a better preventive approach must continue because large clinical trials have consistently demonstrated a drop in the rate of ROP if a lower oxygen saturation target was maintained, but at the expense of an increased rate of neonatal death [59,60]. We suggest that clinical strategies aimed at addressing the inflammatory component of the disease in concert with managing oxidative stress and angiogenesis may prevent or ameliorate long-term consequences of ROP, IVH, and PVL [64].

We found NEC and BPD were not significantly impacted by antenatal inflammation. Although in pregnancies complicated by IAI we cannot exclude a relationship between antenatal risk factors (i.e. genetic susceptibility, FGR) and BPD, our results imply that lung immaturity, reflected in the need for ventilator support and surfactant are the main risk factors for BPD [65–67]. Similar to us, Stafford et al. showed that infectious morbidities including chorioamnionitis and intrapartum antibiotic administration were not significantly associated with higher NEC [68]. Hackam et al. showed that amniotic fluid can inhibit intestinal mucosa Toll-like receptor-4 signaling within the fetal intestine and attenuate experimental NEC in mice [69]. This protective effect was attributed to epidermal growth factor. Postnatal gut colonization and microbiota, intestinal ischemia, and enteral feeding could play a more important role than antenatal factors [70].

Comparing neonatal outcomes between preterm infants born in the setting of PE vs spontaneous PTB, Bastek et al. found that outcomes differed across GA; outcomes were less favorable for spontaneous PTB infants at earlier GAs (24 to 28 weeks), whereas neonates born to mothers with PE suffered increased morbidity 32 to 34 weeks GA [71]. In contrast to our analysis, this earlier study addressed limited neonatal outcomes, which did not include BPD, ROP, PVL, and death, using two non-contemporaneous cohorts. Due to rapid advancement in neonatal care, differences in neonatal management may have contributed to their findings.

The main strength of our study was completeness of data in a cohort of neonates whose mothers were followed prospectively following either a clinically indicated amniocentesis to rule-out IAI, or iatrogenic delivery for PE. In comparison to many epidemiologic studies, our analysis, for the first time, includes assessment of cord blood and amniotic fluid biomarkers of inflammation. As such, inflammatory status of the fetal and gestational sac compartments was addressed objectively, and not subjectively as is often the case when maternal clinical chorioamnionitis is reported [20]. The findings of this study should be viewed in the context of the impossibility to address all the components of the exposome including genetic susceptibility for ROP, BPD, LOS, and possibly NEC and IVH [72–76]. Based on inherent complexity, the possibility of residual confounding in our analyses cannot be excluded, and no causal inferences can be made at this point. Neonates born late preterm (34–37 weeks) have an increased risk of morbidity [38]. At our institution, an amniocentesis to rule-out IAI is not recommended after 34 weeks GA. In addition, delivery of the patients presenting with PPROM after 34 weeks is routinely indicated immediately following confirmation of the diagnosis [77]. Therefore, in these clinical scenarios, evaluation of IAI as a risk factor for IVH, PVL, ROP, death, EONS, NEC, and BPD would have been difficult to assess, especially when the prevalence of these outcomes is low after 34 weeks of gestation.

## Conclusions

When prematurity is unavoidable, antenatal and postnatal risk factors play distinct roles in the development of IVH, NEC, PVL, BPD, ROP, EONS, and LOS. Our data offer an opportunity to better counsel mothers at risk for spontaneous or iatrogenic prematurity in the setting of IAI or PE. Prevention of IVH, NEC, PVL, BPD, ROP, EONS, and LOS must focus on identification and differential targeting of specific antenatal, perinatal, and postnatal events.

## Supporting information

S1 Appendix Supplemental methods. (PDF)
