# Supplementary material for: Components of the antepartum, intrapartum, and postpartum exposome impact on distinct short-term adverse neonatal outcomes of premature infants: A prospective cohort study
Source: PLoS One. 2018 Dec 5;13(12):e0207298. doi: 10.1371/journal.pone.0207298 (PMC6281222; doi:10.1371/journal.pone.0207298)
Supplement: S1 Appendix — (PDF) [file pone.0207298.s001.pdf]

COMPONENTS OF THE ANTEPARTUM, INTRAPARTUM AND POSTPARTUM EXPOSOME IMPACT ON DISTINCT SHORT-TERM ADVERSE NEONATAL OUTCOMES OF PREMATURE INFANTS: A PROSPECTIVE COHORT STUDY

Unzila Ali Nayeri M.D., Catalin S. Buhimschi M.D., MBA, Guomao Zhao B.S.,
Irina A. Buhimschi M.D., M.M.S., Vineet Bhandari M.D., D.M.

S1 Appendix.

Supplemental Methods

a) Definition of Early-Onset Neonatal Sepsis (EONS)

b) Clinical Diagnosis and Research Evaluation of Intra-amniotic-Inflammation/Infection

c) Preparation and Storage of Amniotic Fluid and Cord Blood

d) Placental Evaluation for Histologic Chorioamnionitis

e) Proteomic profiling of amniotic fluid

f) Interleukin-6 (IL-6) Immunoassays

g) Assessment of Fetal Exposure to Intra-amniotic-Inflammation/Infection by Haptoglobin switch-on status

Supplemental References

Supplemental Methods

a) Definition of Early-Onset Neonatal Sepsis (EONS). EONS was defined as confirmed or suspected sepsis at ≤72 hours after birth [1]. Neonatal hematological indices and sepsis categorization were assessed from neonatal blood specimens and microbial cultures obtained within 2h from birth. Confirmed sepsis referred to a positive blood culture. Neonatal blood was collected in a sterile fashion from 2 separate sites prior to antibiotic treatment, and within 2h of birth. A diagnosis of suspected EONS was based on clinical symptoms with specific hematological laboratory results [1]. The following hematological criteria were used as indicators of EONS: **i)** absolute neutrophil count of <7,500 or >14,500 cells/mm^3; **ii)** absolute band count >1,500 cells/mm^3; **iii)** immature/total neutrophil (I:T) ratio >0.16; **iv)** platelet count <150,000 cells/mm^3 [2]. EONS was suspected in the presence of ≥2 hematological criteria in the absence of a positive blood culture. EONS was dichotomized into present (sepsis either confirmed or suspected) or absent. All neonates with confirmed or suspected EONS received broad spectrum antibiotic therapy, per institutional protocol.

b) Clinical Diagnosis and Research Evaluation of Intra-amniotic-Inflammation/Infection (IAI). Laboratory tests performed for the purpose of diagnosing IAI included glucose, lactate dehydrogenase (LDH), Gram stain and white blood cell (WBC) count. For clinical management, an amniotic fluid glucose cut-off of ≤15 mg/dL and LDH levels ≥419 U/L were considered suggestive of intra-amniotic infection [3,4]. Microbiological analysis of amniotic fluid was performed immediately following collection. Presence of microorganisms was assessed using Gram stain and traditional microbial culturing method techniques. Briefly, amniotic fluid was cultured for aerobic and anaerobic bacteria, and *Ureaplasma* and *Mycoplasma* spp.. The results of the microbiological tests were available for case management and were reported as final after 5 days of culturing. Presumptive identification was based on standard microbiological criteria of colony morphology, medium reaction, Gram stain, and the use of a VITEK 2 automated card system (bioMérieux, Hazelwood, MO, http://www.biomerieux-usa.com) for microbiological identification based on biochemical tests and antibiotic susceptibility.

c) Preparation and Storage of Amniotic Fluid and Cord Blood. After collection amniotic fluid and cord blood samples were centrifuged at 1,000×*g* for 15 minutes. The amniotic fluid supernatant and cord blood serum were aliquoted in sterile polypropylene tubes and stored at -80°C for research analyses.

d) Placental Evaluation for Histologic Chorioamnionitis (HCA). In all 378 cases, hematoxylin and eosin- stained sections of extraplacental membranes (amnion and chorio-decidua), chorionic plate, chorio-decidua, and umbilical cord were examined systematically for inflammation. Three stages of HCA (stage I: intervillositis, stage II: chorionic inflammation, and stage III: full-thickness inflammation of both chorion and amnion) were complemented by a previously described histological grading system that includes 4 grades of inflammation of the amnion, chorio-decidua, and umbilical cord [5]. Maternal HCA was defined as absent (amnionitis grade 0 = 0), mild (amnionitis grades 1-2 = 1), or severe (amnionitis grades 3-4 = 2) [6]. A similar scoring system was used for fetal HCA: absent (chorionic plate 0 and funisitis 0 = 0), mild (chorionic plate I-II and funisitis grades 1-2), or severe (chorionic plate III and funisitis grades 3-4).

e) Proteomic profiling of amniotic fluid. Presence of 3 or 4 of the following biomarkers established the diagnosis of IAI: defensin-1, defensin-2, calgranulin-A, calgranulin-C [7,8]. A value of 1 was assigned if a biomarker peak was present and 0 if absent. The “severity” of inflammation was reported as follows: MR 0: “no” inflammation; MR 1-2: “minimal” inflammation; MR 3-4: “severe” inflammation).

f) Interleukin-6 (IL-6) Immunoassays. All amniotic fluid and cord blood samples were assayed in duplicate and dilution factor was varied for correct interpolation within the standard curve. The minimal detectable concentration was 1 pg/mL and the inter- and intra-assay coefficients of variation were <10%.

g) Assessment of Fetal Exposure to Intra-amniotic-Inflammation/Infection (IAI) by Haptoglobin (Hp) switch-on status. Hp is a tetrameric protein with two α and two β-chains. The human population has three major Hp phenotypes (Hp1-1, Hp2-2 and the heterozygous Hp1-2), derived from variations in the α-chain with identical β-chains. Absence of Hp at the protein level denotes Hp0-0 phenotype (anhaptoglobinemia). A detectable Hp β-chain (42 kDa) on Western blot was indicative of a “switch-on” in Hp expression. Hp phenotypes were identified by additional presence of α chain bands at either ~9 kDa (α^1: Hp1-1), ~20 kDa (α^2: Hp2-2) or both (Hp1-2). To assess for fetal exposure to IAI, we first screened all cord blood serum samples for Hp immunoreactivity by ELISA (US Biological, Swampscott, MA). Western blotting with polyclonal anti-Hp antibody (Sigma, St Louis, MO) was used to confirm Hp switch-on status and to assign the Hp phenotype as previously described.

Supplemental References

1. Rodwell RL, Leslie AL, Tudehope DI. Early diagnosis of neonatal sepsis using a hematologic scoring system. J Pediatr. 1988;112(5): 761-767.

2. Bhandari V, Wang C, Rinder C, Rinder H. Hematologic profile of sepsis in neonates: Neutrophil CD64 as a diagnostic marker. Pediatrics. 2008;121(1): 129-134.

3. Edwards RK, Clark P, Locksmith Gregory J, Duff P. Performance characteristics of putative tests for subclinical chorioamnionitis. Infect Dis Obstet Gynecol. 2001;9: 209-214.

4. Garry D, Figueroa R, Aguero Rosenfeld M, Martinez E, Visintainer P, Tejani N. A comparison of rapid amniotic fluid markers in the prediction of microbial invasion of the uterine cavity and preterm delivery. Obstet Gynecol. 1996;175: 1336-1341.

5. Salafia CM, Weigl C, Silberman L. The prevalence and distribution of acute placental inflammation in uncomplicated term pregnancies. Obstet Gynecol. 1989;73(3): 383-389.

6. Ghidini A, Salafia CM, Kirn V, Doria V, Spong CY. Biophysical profile in predicting acute ascending infection in preterm rupture of membranes before 32 weeks. Obstet Gynecol. 2000;96(2): 201-206.

7. Buhimschi I, Christner R, Buhimschi C. Proteomic biomarker analysis of amniotic fluid for identification of intra-amniotic inflammation. BJOG. 2005;112(2): 173-181.

8. Buhimschi CS, Bhandari V, Hamar BD, Bahtiyar MO, Zhao G, Sfakianaki AK, et al. Proteomic profiling of the amniotic fluid to detect inflammation, infection, and neonatal sepsis. PLoS Med. 2007;4(1): e18.
